# Association of low-carbohydrate-diet score and cognitive performance in older adults: National Health and Nutrition Examination Survey (NHANES)

**DOI:** 10.1186/s12877-022-03607-1

**Published:** 2022-12-20

**Authors:** Huiqin Wang, Yan Lv, Gang Ti, Gang Ren

**Affiliations:** 1 Department of Neurology, Shanxi Bethune Hospital, Shanxi Academy of Medical Sciences, Tongji Shanxi Hospital, Third Hospital of Shanxi Medical University, No.99 Longcheng Street, 030032 Taiyuan, Shanxi Province, People’s Republic of China; 2 Department of Nephrology, Shanxi Bethune Hospital, Shanxi Academy of Medical Sciences, Tongji Shanxi Hospital, Third Hospital of Shanxi Medical University, 030032 Taiyuan, Shanxi Province, People’s Republic of China; 3 Department of Medical Record, Shanxi Bethune Hospital, Shanxi Academy of Medical Sciences, Tongji Shanxi Hospital, Third Hospital of Shanxi Medical University, 030032 Taiyuan, Shanxi Province, People’s Republic of China

**Keywords:** Low-carbohydrate-diet score, Cognitive performance, Older adults, NHANES

## Abstract

**Background:**

To investigate the association between low-carbohydrate-diet (LCD) score and cognitive performance based on a nationally representative sample aged ≥ 60 years from National Health and Nutrition Examination Survey (NHANES) database.

**Methods:**

This cross-sectional study included 2,537 eligible older adults from the NHANES database 2011–2014. The Consortium to Establish a Registry for Alzheimer’s Disease (CERAD) word learning subtest, Animal Fluency Test (AFT), and Digit Symbol Substitution Test (DSST) were used to assess the cognitive performance. All participants were categorized into the low and normal cognitive performance groups. The univariate and multivariate logistic regression analyses were utilized to evaluate the association of LCD score with cognitive performance. Stratified analyses based on age, body mass index (BMI), gender, marital status, education level was conducted.

**Results:**

After adjusting age, education level, marital status, household income, history of diabetes, history of hypertension, history of congestive heart failure, history of coronary heart disease, history of heart disease, history of stroke, magnesium and the using of psychotropic medication, LCD score was correlated with the CERAD word learning subtest. The associations between LCD score and AFT, DSST were not statistically significant. Moreover, LCD score was also related to cognitive performance among individuals who were aged < 65 years or BMI 25–30 kg/m^2^ or was married/separated, or had an education level of high school or above.

**Conclusion:**

The adherences to LCD might be associated with the risk of cognitive performance among older adults. Further large-scale cohort studies are needed to test the causal relationship of LCD and cognitive performance.

**Supplementary Information:**

The online version contains supplementary material available at 10.1186/s12877-022-03607-1.

## Background

Nowadays, global aging is increasing as life expectancy increases, and at the same time, cognitive decline related to age may be a primary health issue for elderly population [1]. It is estimated that the number of people in the United States suffered from cognitive impairment increased from 12.23 million in 2020 to 21.55 million in 2060 [2]. The irreversibility of cognitive impairment made the prevention and treatment of low cognitive performance a top priority [3]. Therefore, it is important to explore modifiable lifestyle and some risk factors to prevent low cognitive performance.

Diet is a kind of modifiable factor. It has previously been proposed that diet might play an important role in the intervention strategy for cognitive performance [4, 5]. Fan, et al. reported that adherence to higher Dietary Guidelines for Americans (DGA) was associated with a better cognitive performance (such as processing speed and executive function) for American adults aged ≥ 60 years [6]. Carbohydrates, fats, and proteins are considered as the important nutrients required for brain health, which has received much attention in a healthy diet [7]. Carbohydrates are the main source of energy in the diet and consumed mainly through foods such as rice, potatoes and grains [8]. In the study of Muth AK et al., they pointed out that carbohydrate consumption was associated with the cognitive function [7]. Additionally, Li Y, et al. found that dietary protein intake was positively correlated with cognitive function in older adults [9]. In the recent years, a low-carbohydrate-diet (LCD) score characterized by a diet with lower intake of carbohydrates and higher intakes of proteins and fats has been proposed [10]. LCD score has received widespread attention as a viable option for the treatment of losing weight and preventing obesity [11, 12]. Previous studies have showed that LCD score was linked to many aspects of health, such as diabetes mellitus, coronary artery calcium progression, psychological disorders, and so on [10, 13, 14]. Sangsefidi, et al. reported that LCD score might be associated with lower chance of metabolic syndrome in Iranian adults based on a cross-sectional study [11]. However, to the best of our knowledge, few studies have explored the associations of LCD score and cognitive performance in the older adults so far.

Our hypothesis in this study is that LCD score may be associated with cognitive performance for older adults due to oxidative stress and inflammation. We investigated the association of LCD score and cognitive performance based on a nationally representative sample of aged ≥ 60 years from National Health and Nutrition Examination Survey (NHANES) database.

## Methods

### Data sources and study population

In this cross-sectional study, all information of study population derived from the NHANES database. The NHANES data is a representative sample of the non-institutionalized population of the USA. It is a two-year-cycle program performed by the Centers for Disease Control and Prevention (CDC) of America using a multistage, probability sampling methods [1, 15]. NHANES examines a nationally representative sample of approximately 5,000 people from 15 different counties each year [3]. Its primary goal is to assess the health and nutritional status of adults and children in the United States. CDC obtained ethical approval and consented all the participants.

**Fig. 1:**
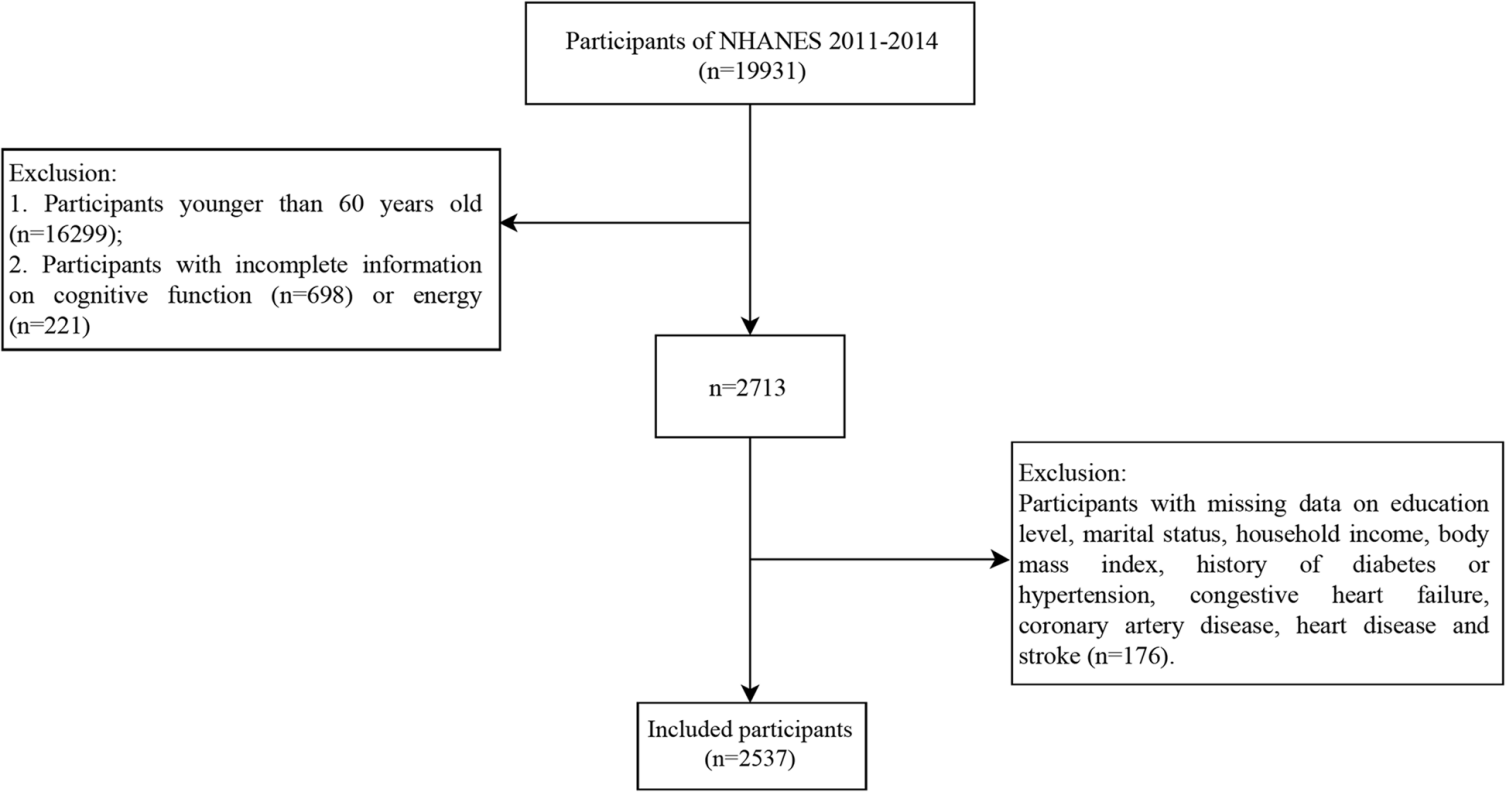
Flowchart showing the selection of study population

In the current study, we selected two NHANES cycles, namely, NHANES 2011–2012 and 2013–2014; because some cognitive function tests were specially conducted in these two NHANES cycles. A total of 19,931 people participated in the NHANES from 2011–2014. Our study was limited to 2,731 adults aged 60 years or older with complete information on cognitive function and energy. Among them, we excluded some individuals with missing information on education level, marital status, household income, body mass index (BMI), history of diabetes or hypertension, congestive heart failure, coronary artery disease, heart disease and stroke. Finally, 2,537 eligible older adults were included (Fig. 1). This study only obtained public data, so ethical review was not required by Shanxi Bethune Hospital, Shanxi Academy of Medical Sciences, Tongji Shanxi Hospital, Third Hospital of Shanxi Medical University.

### Data collection

The following variables were extracted in this study [1, 16]: age (years), gender, race, education level, marital status, household income (5$), BMI (kg/m^2^), waistline (cm), history of diabetes, history of hypertension, history of congestive heart failure, history of coronary heart disease, history of heart disease, history of stroke, magnesium (mg), calcium (mg), vitamin D (mcg), the using of psychotropic medication, carbohydrate intake score, fat intake score, protein intake score. BMI was calculated as weight divided by height squared. History of diabetes or hypertension was defined as self-reported physician diagnosis.

### Dietary assessment

All NHANES participants were asked to undergo a first dietary recall interview at the Mobile Examination Center, and a second interview data via telephone 3 to 10 days later [17]. For participants of NHANES database, dietary intake data were used to estimate the types and amounts of foods and beverages consumed during the 24‐hour period before the interview (midnight to midnight), and to estimate the energy, nutrition, and other food components consumed from these foods and beverages. In this study, dietary intake was estimated using the average of data from two 24-h dietary recall.

### Calculation of the low-carbohydrate-diet score

The average dietary data from two 24-h dietary recall interview was used to calculate the intakes of fat, protein, carbohydrate and energy. LCD score was calculated based on a comprehensive assessment of fat, protein and carbohydrate [12]. The intakes per gram of fat, protein, and carbohydrate were first converted to kilocalories (conversion ratio: 1:9, 1:4, and 1:4, respectively); then we calculated the percentage of carbohydrate (kilocalories) and total energy, the percentage of protein (kilocalories) and total energy, and the percentage of fat (kilocalories) and total energy. For fat and protein, the highest percentage of intake was scored 10 points, and the lowest was 0 points. For carbohydrates, those with the lowest percentage of intake scored 10 points, and the highest intake scored 0 points (Supplemental Table 1). LCD score is the sum of the scores for the three nutrients, ranging from 0 to 30. The higher score reflects a higher intake of fat and protein and a lower intake of carbohydrate. In this study, LCD score was divided into five groups according to 20% quantiles, 40% quantiles, 60 quantiles and 80% quantiles: < 4 points group; 4–8 points group; 8–12 points group; 12–17 points group and > 17 points group.

**Table 1:** The characteristics of all eligible participants

Variables	CERAD word learning subtest			AFT			DSST		
	**Normal cognitive performance group (*N* = 1526)**	**Low cognitive performance group (*N* = 1011)**	***P***	**Normal cognitive performance group (*N* = 1517)**	**Low cognitive performance group (*N* = 1020)**	***P***	**Normal cognitive performance group (*N* = 1535)**	**Low cognitive performance group (*N* = 1002)**	***P***
LCD score, Mean (S.E)	11.38 (0.23)	10.38 (0.29)	0.001	11.16 (0.23)	10.75 (0.36)	0.295	11.18 (0.24)	10.66 (0.34)	0.174
Carbohydrate intake score, Mean (S.E)	3.73 (0.11)	3.32 (0.14)	0.005	3.67 (0.11)	3.43 (0.16)	0.152	3.69 (0.11)	3.36 (0.15)	0.046
Fat intake score, Mean (S.E)	4.63 (0.11)	4.38 (0.15)	0.108	4.52 (0.10)	4.58 (0.16)	0.675	4.58 (0.11)	4.44 (0.16)	0.388
Protein intake score, Mean (S.E)	3.02 (0.10)	2.68 (0.14)	0.038	2.98 (0.09)	2.74 (0.13)	0.122	2.91 (0.09)	2.86 (0.14)	0.757
Gender, n (%)			0.865			< 0.001			0.003
Male	713 (46.09)	532 (46.49)		802 (49.97)	443 (39.23)		755 (48.96)	490 (40.16)	
Female	813 (53.91)	479 (53.51)		715 (50.03)	577 (60.77)		780 (51.04)	512 (59.84)	
Age, years, Mean (S.E)	68.06 (0.21)	70.63 (0.37)	< 0.001	68.43 (0.21)	70.03 (0.35)	< 0.001	68.45 (0.21)	70.18 (0.37)	< 0.001
Race, n (%)			0.255			< 0.001			< 0.001
Mexican American	123 (2.80)	91 (3.70)		143 (3.12)	71 (3.13)		108 (2.27)	106 (5.02)	
Other Hispanic	141 (2.98)	108 (4.13)		147 (3.02)	102 (4.08)		109 (2.13)	140 (6.21)	
Non-Hispanic White	743 (81.45)	526 (80.41)		806 (83.69)	463 (76.17)		846 (84.58)	423 (73.27)	
Non-Hispanic Black	370 (7.84)	222 (7.83)		304 (6.08)	288 (11.14)		306 (5.84)	286 (12.29)	
Other Race: Including Multi-Racial	149 (4.93)	64 (3.93)		117 (4.09)	96 (5.48)		166 (5.18)	47 (3.22)	
Education, n (%)			< 0.001			< 0.001			< 0.001
Less than high school	269 (10.33)	339 (23.47)		283 (10.84)	325 (23.01)		185 (8.54)	423 (29.61)	
High school and above	1257 (89.67)	672 (76.53)		1234 (89.16)	695 (76.99)		1350 (91.46)	579 (70.39)	
Marital status, n (%)			0.008			0.007			< 0.001
Married	870 (65.58)	544 (59.55)		876 (65.64)	538 (59.22)		917 (67.10)	497 (55.16)	
Divorced	244 (13.72)	226 (19.65)		243 (13.71)	227 (19.89)		235 (13.23)	235 (21.71)	
Separated	249 (13.25)	125 (11.92)		226 (12.79)	148 (12.74)		228 (12.57)	146 (13.21)	
Other^a^	163 (7.45)	116 (8.88)		172 (7.87)	107 (8.15)		155 (7.09)	124 (9.92)	
Household income, $, Mean (S.E)	48,459.93 (1147.74)	40,649.94 (1155.73)	< 0.001	48,165.23 (1176.77)	40,912.60 (1118.19)	< 0.001	48,623.77 (1220.00)	39,005.55 (1162.59)	< 0.001
BMI, kg/m^2^, Mean (S.E)	28.95 (0.24)	29.38 (0.31)	0.125	28.85 (0.21)	29.59 (0.36)	0.023	28.83 (0.24)	29.71 (0.38)	0.039
Waistline, cm, Mean (S.E)	101.99 (0.61)	103.49 (0.84)	0.102	102.22 (0.53)	103.13 (0.90)	0.272	102.11 (0.61)	103.50 (1.00)	0.211
Diabetes, n (%)			0.037			< 0.001			< 0.001
Yes	322 (17.39)	260 (21.94)		309 (16.47)	273 (23.84)		291 (16.01)	291 (25.76)	
No	1132 (78.47)	705 (73.74)		1133 (79.24)	704 (72.11)		1169 (79.81)	668 (69.99)	
Borderline	72 (4.14)	46 (4.32)		75 (4.28)	43 (4.05)		75 (4.18)	43 (4.25)	
Hypertension, n (%)			< 0.001			< 0.001			< 0.001
Yes	902 (53.11)	676 (66.66)		875 (53.72)	703 (66.02)		889 (52.99)	689 (69.15)	
No	624 (46.89)	335 (33.34)		642 (46.28)	317 (33.98)		646 (47.01)	313 (30.85)	
Congestive heart failure, n (%)			< 0.001			< 0.001			< 0.001
Yes	71 (4.22)	102 (10.60)		77 (4.61)	96 (10.10)		74 (4.51)	99 (11.00)	
No	1455 (95.78)	909 (89.40)		1440 (95.39)	924 (89.90)		1461 (95.49)	903 (89.00)	
Coronary heart disease, n (%)			0.003			0.036			0.052
Yes	113 (7.56)	120 (13.12)		125 (8.40)	108 (11.76)		127 (8.47)	106 (12.00)	
No	1413 (92.44)	891 (86.88)		1392 (91.60)	912 (88.24)		1408 (91.53)	896 (88.00)	
Heart disease, n (%)			< 0.001			0.040			< 0.001
Yes	120 (7.41)	100 (10.92)		122 (7.82)	98 (10.29)		110 (7.23)	110 (11.90)	
No	1406 (92.59)	911 (89.08)		1395 (92.18)	922 (89.71)		1425 (92.77)	892 (88.10)	
Stroke, n (%)			< 0.001			0.005			< 0.001
Yes	75 (4.73)	91 (7.97)		73 (4.83)	93 (7.91)		66 (4.31)	100 (9.44)	
No	1451 (95.27)	920 (92.03)		1444 (95.17)	927 (92.09)		1469 (95.69)	902 (90.56)	
Magnesium, mg, Mean (S.E)	305.39 (6.22)	269.36 (5.16)	< 0.001	303.64 (5.88)	266.84 (6.86)	< 0.001	306.24 (6.48)	264.74 (5.46)	< 0.001
Calcium, mg, Mean (S.E)	915.89 (20.49)	858.58 (20.75)	0.029	912.27 (20.29)	858.26 (24.52)	0.081	914.23 (20.80)	861.38 (24.13)	0.087
Vitamin D, mcg, Mean (S.E)	5.05 (0.17)	4.84 (0.22)	0.511	5.00 (0.14)	5.00 (0.31)	0.994	4.98 (0.17)	5.07 (0.28)	0.803
Psychotropic medication, n (%)			< 0.001			< 0.001			< 0.001
No	1346 (99.79)	777 (97.76)		1318 (99.78)	805 (97.75)		1346 (99.75)	777 (97.51)	
Yes	3 (0.21)	16 (2.24)		2 (0.22)	17 (2.25)		4 (0.25)	15 (2.49)	

**Note:** Other^a^, represents widowed, never married and living with partner; *BMI* Body mass index, *CERAD*, Consortium to Establish a Registry for Alzheimer’s Disease, *AFT* Animal Fluency Test, *DSST* Digit Symbol Substitution Test

### Cognitive performance assessment

A series of cognitive function testing in the adults aged ≥ 60 years from NHANES were used in the 2011–2014, including the Consortium to Establish a Registry for Alzheimer’s Disease (CERAD) word learning subtest, Animal Fluency Test (AFT), Digit Symbol Substitution Test (DSST) [1].

CERAD word learning subtest [6], which consists of three consecutive learning trials as well as a delayed recall trial, is used to evaluate immediate and delayed memory. In the learning trials, participants were asked to read 10 unrelated words one at a time. After completing the DSST and AFT assessments, the delayed recall trial asked participants to recall 10 words used in the learning experiment. With scores ranging from 0 to 10 for each trial, and the final score of the CERAD test was the sum of three consecutive learning trials and a delayed recall trial.

AFT [1], as a component of executive function, is performed to assess the categorical verbal fluency. Participants were asked to name as many animals as possible in one minute with each named animal receiving one point, and the final score of AFT was the sum for the number of correctly named animals.

DSST [15] is used to assess processing speed, sustained attention and working memory. The test was performed by using a paper form with a key at the top pairing numbers with nine symbols. Participants were asked to match the corresponding symbols from the 133 boxes that held adjacent numbers in two minutes. The final score of DSST was the sum for the number of correct matches.

The outcome of this study was the cognitive performance, which contained CERAD word learning subtest, AFT, and DSST. Currently, there is no standard for the CERAD word learning subtest, AFT, and DSST tests to identify low cognitive performance. Therefore, we used the lowest quartile as the cut-off point; below or equal to the lower quartile was considered as low cognitive population, and above the lower quartile was normal population, which was also consistent with some literature [1, 18].

### Statistical analysis

In the present study, we described the normally distributed variables by mean ± standard error (mean ± SE), and comparison between the low cognitive performance group and the normal cognitive performance group adopted Student’s t-test. The number of cases and the composition ratio [n (%)] was used to describe the categorical data, and comparison between two groups adopted χ^2^ test. “n” was considered as unweighted number, and “(%)” was considered as weighted percentage.

For the current study, all participants were categorized into the low cognitive performance group and the normal cognitive performance group. We conducted a descriptive analysis between two groups. Then we conducted the univariate and multivariate logistic regression analyses to examine the association between LCD score and cognitive performance. We adopted restricted cubic spline (RCS) curves to assess the dose–response relationship of LCD score and cognitive performance. Additionally, we also performed stratified analyses based on age, BMI, gender, marital status, education level to further examine the associations between LCD score and cognitive performance. The missing values were deleted in this study, and the proportion of missing values was shown in the Supplemental Table 2. In addition, we also performed the sensitivity analysis before and after deletion of missing values (Supplemental Table 3). SAS 9.4 and R 4.0.3 software were used for statistical analyses, and we calculated odds ratio (OR) and 95% confidence interval (CI). *P* < 0.05 was considered as statistically significant difference.

**Table 2:** The association between LCD score and cognitive performance

Variables	LCD score level	Model 1		Model 2		Model 3	
		**OR (95%CI)**	***P***	**OR (95%CI)**	***P***	**OR (95%CI)**	***P***
**CERAD word learning subtest **^**#**^	< 4	Ref		Ref		Ref	
	4–8	0.58 (0.43–0.78)	0.001	0.59 (0.44–0.80)	0.003	0.56 (0.42–0.75)	0.003
	8–12	0.58 (0.44–0.78)	0.001	0.58 (0.43–0.79)	0.002	0.60 (0.42–0.86)	0.019
	12–17	0.49 (0.33–0.71)	< 0.001	0.53 (0.37–0.77)	0.003	0.56 (0.38–0.82)	0.014
	> 17	0.68 (0.50–0.91)	0.016	0.74 (0.55–1.01)	0.071	0.78 (0.55–1.10)	0.185
AFT*	< 4	Ref		Ref		Ref	
	4–8	0.72 (0.53–0.97)	0.041	0.76 (0.55–1.04)	0.098	0.72 (0.52–1.00)	0.186
	8–12	0.65 (0.47–0.90)	0.014	0.68 (0.48–0.95)	0.034	0.70 (0.48–1.03)	0.213
	12–17	0.61 (0.46–0.81)	0.002	0.70 (0.53–0.93)	0.023	0.73 (0.54–0.98)	0.170
	> 17	0.84 (0.57–1.23)	0.374	0.96 (0.65–1.43)	0.856	1.00 (0.65–1.52)	0.984
**DSST**^**&**^	< 4	Ref		Ref		Ref	
	4–8	0.72 (0.53–0.97)	0.041	0.76 (0.55–1.04)	0.098	0.72 (0.52–1.00)	0.186
	8–12	0.65 (0.47–0.90)	0.014	0.68 (0.48–0.95)	0.034	0.70 (0.48–1.03)	0.213
	12–17	0.61 (0.46–0.81)	0.002	0.70 (0.53–0.93)	0.023	0.73 (0.54–0.98)	0.170
	> 17	0.84 (0.57–1.23)	0.374	0.96 (0.65–1.43)	0.856	1.00 (0.65–1.52)	0.984

**Note:** *LCD* Low-carbohydrate-diet, *CERAD* Consortium to Establish a Registry for Alzheimer’s Disease, *AFT* Animal Fluency Test, *DSST* Digit Symbol Substitution Test, *OR* Odds ratio, *CI* Confidence interval

Model 1, did not adjust for any confounders;

Model 2, adjusted for age, gender and race;

Model 3 (for CERAD word learning subtest ^#^), adjusted for age, education level, marital status, household income, history of diabetes, hypertension, congestive heart failure, coronary heart disease, heart disease and stroke, magnesium and the using of psychotropic medication;

Model 3 (for AFT*), adjusted for age, gender, race, education level, marital status, household income, body mass index; history of diabetes, hypertension, congestive heart failure, coronary heart disease, heart disease and stroke, magnesium, calcium and the using of psychotropic medication;

Model 3 (for DSST^&^), adjusted for age, gender, race, education level, marital status, household income, body mass index; history of diabetes, hypertension, congestive heart failure, coronary heart disease, heart disease and stroke, magnesium and calcium

**Table 3:** Stratified analyses by age, BMI, gender, marital status and education level

Variables		LCD score level		CERAD word learning subtest ^#^		AFT*		DSST^&^	
				**OR (95%CI)**	***P***	**OR (95%CI)**	***P***	**OR (95%CI)**	***P***
**Age, years**									
60 ≤ and < 65		< 4		Ref		Ref		Ref	
		4–8		0.22 (0.09–0.52)	0.005	0.45 (0.24–0.85)	0.071	0.39 (0.20–0.74)	0.045
		8–12		0.37 (0.19–0.71)	0.012	0.57 (0.25–1.31)	0.255	0.56 (0.24–1.30)	0.247
		12–17		0.42 (0.20–0.89)	0.043	0.88 (0.42–1.81)	0.740	0.65 (0.32–1.31)	0.292
		> 17		0.49 (0.20–1.20)	0.145	1.07 (0.54–2.13)	0.850	1.00 (0.45–2.20)	0.994
≥ 65		< 4		Ref		Ref		Ref	
		4–8		0.78 (0.54–1.11)	0.194	0.83 (0.60–1.15)	0.341	0.81 (0.52–1.26)	0.410
		8–12		0.71 (0.48–1.05)	0.115	0.71 (0.48–1.06)	0.190	0.68 (0.48–0.96)	0.115
		12–17		0.59 (0.36–0.96)	0.059	0.64 (0.44–0.92)	0.097	0.63 (0.40–0.98)	0.133
		> 17		0.84 (0.61–1.15)	0.299	0.94 (0.61–1.46)	0.797	0.95 (0.57–1.56)	0.848
**Gender**									
Male		< 4		Ref		Ref		Ref	
		4–8		0.37 (0.21–0.64)	0.005	0.74 (0.43–1.30)	0.356	0.80 (0.46–1.40)	0.486
		8–12		0.54 (0.29–1.00)	0.075	0.70 (0.39–1.24)	0.289	0.67 (0.36–1.25)	0.276
		12–17		0.54 (0.33–0.88)	0.031	0.77 (0.51–1.16)	0.275	0.77 (0.48–1.26)	0.358
		> 17		0.82 (0.48–1.40)	0.483	1.30 (0.72–2.37)	0.434	1.54 (0.89–2.67)	0.194
Female		< 4		Ref		Ref		Ref	
		4–8		0.73 (0.50–1.07)	0.139	0.71 (0.49–1.04)	0.175	0.57 (0.36–0.92)	0.105
		8–12		0.67 (0.42–1.07)	0.127	0.75 (0.45–1.26)	0.357	0.65 (0.42–1.00)	0.146
		12–17		0.53 (0.36–0.79)	0.010	0.71 (0.47–1.08)	0.204	0.55 (0.33–0.92)	0.106
		> 17		0.70 (0.43–1.13)	0.172	0.79 (0.46–1.37)	0.465	0.69 (0.42–1.13)	0.233
**BMI, kg/m**^**2**^									
≤ 25	< 4		Ref			Ref		Ref	
	4–8		1.13 (0.58–2.21)		0.726	0.97 (0.55–1.71)	0.924	0.97 (0.44–2.15)	0.941
	8–12		0.95 (0.43–2.07)		0.892	0.78 (0.43–1.40)	0.447	0.88 (0.38–2.03)	0.774
	12–17		0.61 (0.29–1.28)		0.218	0.44 (0.20–0.98)	0.116	0.78 (0.36–1.69)	0.561
	> 17		1.53 (0.76–3.09)		0.259	1.12 (0.55–2.26)	0.768	1.33 (0.59–3.01)	0.535
> 25 and ≤ 30	< 4		Ref			Ref		Ref	
	4–8		0.34 (0.18–0.63)		0.006	0.73 (0.43–1.23)	0.325	0.43 (0.23–0.81)	0.079
	8–12		0.39 (0.21–0.73)		0.014	0.53 (0.31–0.90)	0.102	0.42 (0.24–0.76)	0.062
	12–17		0.38 (0.18–0.80)		0.028	0.86 (0.53–1.40)	0.598	0.50 (0.26–0.94)	0.121
	> 17		0.52 (0.34–0.82)		0.017	0.84 (0.48–1.49)	0.595	0.62 (0.30–1.27)	0.282
> 30	< 4		Ref			Ref		Ref	
	4–8		0.55 (0.29–1.05)		0.102	0.63 (0.38–1.07)	0.185	0.86 (0.50–1.45)	0.604
	8–12		0.61 (0.36–1.04)		0.100	0.77 (0.43–1.40)	0.454	0.78 (0.42–1.46)	0.499
	12–17		0.64 (0.38–1.06)		0.115	0.80 (0.46–1.40)	0.492	0.83 (0.44–1.57)	0.614
	> 17		0.70 (0.36–1.35)		0.316	1.10 (0.64–1.91)	0.756	1.34 (0.74–2.43)	0.406
**Education**									
Less than high school	< 4		Ref			Ref		Ref	
	4–8		0.60 (0.37–0.99)		0.065	0.85 (0.52–1.40)	0.555	0.77 (0.36–1.63)	0.513
	8–12		1.18 (0.63–2.22)		0.618	0.77 (0.51–1.14)	0.239	0.89 (0.43–1.86)	0.772
	12–17		0.88 (0.54–1.45)		0.631	0.42 (0.26–0.69)	0.013	0.68 (0.36–1.28)	0.275
	> 17		1.61 (0.88–2.97)		0.147	0.63 (0.35–1.13)	0.170	0.77 (0.28–2.14)	0.629
High school or above	< 4		Ref			Ref		Ref	
	4–8		0.55 (0.37–0.80)		0.008	0.68 (0.48–0.97)	0.079	0.65 (0.40–1.04)	0.122
	8–12		0.51 (0.34–0.77)		0.007	0.67 (0.43–1.03)	0.119	0.63 (0.39–1.02)	0.110
	12–17		0.50 (0.31–0.80)		0.012	0.77 (0.54–1.11)	0.212	0.65 (0.41–1.04)	0.125
	> 17		0.67 (0.44–1.02)		0.080	1.05 (0.67–1.63)	0.852	1.07 (0.66–1.74)	0.781
**Marital status**									
Married	< 4		Ref			Ref		Ref	
	4–8		0.56 (0.34–0.93)		0.041	0.70 (0.42–1.18)	0.217	0.65 (0.42–1.03)	0.104
	8–12		0.63 (0.40–0.99)		0.061	0.68 (0.44–1.04)	0.115	0.57 (0.35–0.92)	0.050
	12–17		0.62 (0.36–1.06)		0.099	0.89 (0.56–1.39)	0.614	0.76 (0.45–1.28)	0.332
	> 17		0.84 (0.53–1.34)		0.479	1.05 (0.67–1.66)	0.834	0.96 (0.62–1.48)	0.856
Divorced	< 4		Ref			Ref		Ref	
	4–8		0.63 (0.31–1.30)		0.236	0.68 (0.34–1.36)	0.315	0.81 (0.35–1.86)	0.634
	8–12		0.97 (0.47–1.99)		0.930	1.04 (0.43–2.51)	0.929	1.90 (0.91–3.97)	0.138
	12–17		0.54 (0.29–1.02)		0.078	0.79 (0.39–1.61)	0.544	0.71 (0.32–1.60)	0.439
	> 17		0.45 (0.24–0.86)		0.030	0.38 (0.15–0.98)	0.092	0.96 (0.41–2.23)	0.929
Separated	< 4		Ref			Ref		Ref	
	4–8		0.46 (0.20–1.08)		0.095	0.71 (0.28–1.79)	0.490	0.52 (0.25–1.09)	0.127
	8–12		0.29 (0.11–0.74)		0.021	0.40 (0.20–0.82)	0.041	0.29 (0.12–0.71)	0.030
	12–17		0.35 (0.13–0.93)		0.052	0.27 (0.09–0.82)	0.055	0.29 (0.08–1.08)	0.107
	> 17		0.76 (0.23–2.43)		0.645	1.64 (0.55–4.93)	0.405	1.10 (0.33–3.68)	0.876
Other^#^	< 4		Ref			Ref		Ref	
	4–8		1.10 (0.49–2.50)		0.818	0.82 (0.33–2.01)	0.679	1.32 (0.46–3.83)	0.626
	8–12		1.33 (0.52–3.40)		0.558	1.75 (0.73–4.20)	0.267	1.19 (0.41–3.49)	0.758
	12–17		0.73 (0.23–2.28)		0.592	0.23 (0.09–0.57)	0.025	0.30 (0.11–0.86)	0.076
	> 17		0.96 (0.35–2.66)		0.935	0.99 (0.38–2.58)	0.979	0.47 (0.14–1.58)	0.275

**Note:** Other^#^, represents widowed, never married and living with partner; BMI, body mass index; CERAD, Consortium to Establish a Registry for Alzheimer’s Disease; AFT, Animal Fluency Test; DSST, Digit Symbol Substitution Test

For CERAD word learning subtest ^#^: adjusted for age, education level, marital status, household income, history of diabetes, hypertension, congestive heart failure, coronary heart disease, heart disease and stroke, magnesium and the using of psychotropic medication;

For AFT*: adjusted for age, gender, race, education level, marital status, household income, body mass index; history of diabetes, hypertension, congestive heart failure, coronary heart disease, heart disease and stroke, magnesium, calcium and the using of psychotropic medication;

For DSST^&^: adjusted for age, gender, race, education level, marital status, household income, body mass index; history of diabetes, hypertension, congestive heart failure, coronary heart disease, heart disease and stroke, magnesium and calcium

## Results

### Baseline characteristics

The baseline characteristics of all eligible participants were shown in Table 1. We found that there were some significant differences between the low cognitive performance group and the normal cognitive performance group according to CERAD word learning subtest in the distribution of carbohydrate intake score, age, education level, marital status, household income, height, history of diabetes, history of hypertension, history of congestive heart failure, history of coronary heart disease, history of heart disease, history of stroke, magnesium and the using of psychotropic medication. Likewise, there were some differences between the low cognitive performance group and the normal cognitive performance group according to AFT: gender, age, race, education level, marital status, household income, BMI, history of diabetes, history of hypertension, history of congestive heart failure, history of coronary heart disease, history of heart disease, history of stroke, magnesium, calcium and the using of psychotropic medication. Additionally, compared to participants with normal cognitive performance, people with low cognitive performance were more likely to have higher age, lower household income and carbohydrate intake score. The number of the history of hypertension and history of diabetes in people with low cognitive performance was lower than that of people with normal cognitive performance. Detailed information was shown in Table 1.

### The association between LCD score and cognitive performance

**Fig. 2:**
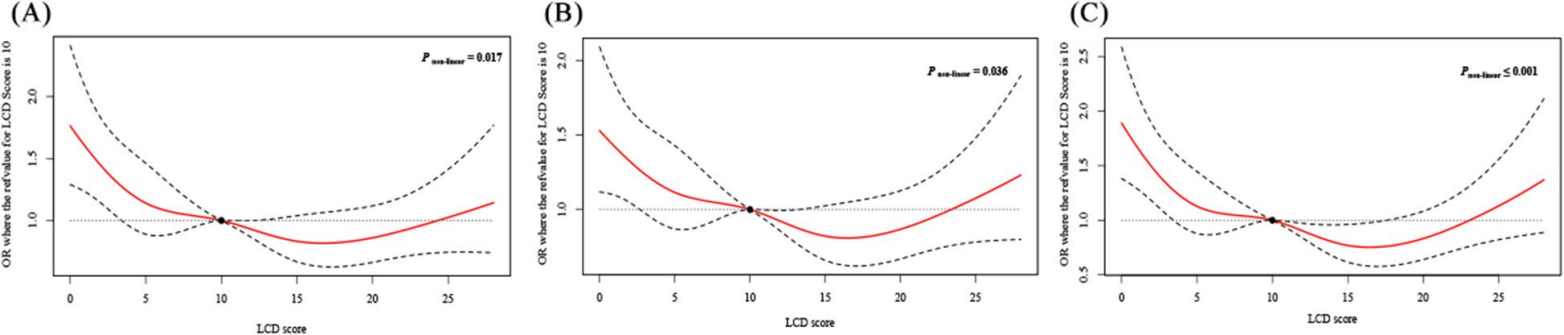
**A** The dose–response relationship between LCD score and CERAD word learning subtest; (**B**) The dose–response relationship between LCD score and AFT; (**C**) The dose–response relationship between LCD score and DSST. The solid line represents the odds ratios, and the dotted line represents the 95% confidence interval; LCD = low-carbohydrate-diet; CERAD = Consortium to Establish a Registry for Alzheimer’s Disease; AFT = Animal Fluency Test; DSST = Digit Symbol Substitution Test

Table 2 displays the association between LCD score and cognitive performance. Three model was used in this study. For the CERAD word learning subtest, after adjusting age, education level, marital status, household income, history of diabetes, history of hypertension, history of congestive heart failure, history of coronary heart disease, history of heart disease, history of stroke, magnesium and the using of psychotropic medication (Model 3), the multivariate logistic regression analysis (Model 3) showed that compared with LCD score < 4 points, OR with 95% CI was 0.56 (0.42–0.75, *P* = 0.003) among LCD score 4–8 points; OR with 95% CI was 0.60 (0.42–0.86, *P* = 0.019) among LCD score 8–12 points; OR with 95% CI was 0.56 (0.38–0.82, *P* = 0.014) among LCD score 12–17 points; OR with 95% CI was 0.78 (0.55–1.10, *P* = 0.185) among LCD score 12–17 points. These results indicated that LCD score was correlated with the CERAD word learning subtest. The associations between LCD score and AFT, DSST were not statistically significant in multivariate logistic regression analysis. Furthermore, the result of RCS curves suggested that there might be a “U-shaped” in the associations of LCD score and cognitive performance (Fig. 2).

### Stratified analyses by age, BMI, gender, marital status and education level

In the present study, we also performed stratified analyses by age, BMI, gender, marital status and education level. The results were shown in Table 3. We found that there was still a correlation between LCD score and the CERAD word learning subtest based on age, BMI, gender, marital status, education level. These results showed that the cognitive performance was associated with LCD score among population who aged < 65 years or BMI 25–30 kg/m^2^ or was married/separated, or had an education level of high school or above, which indicated the association of LCD score and cognitive performance might be more robust for these individuals.

## Discussion

A better understanding of the link between diet and cognitive performance has important implications to prevent and better manage cognitive decline. In this cross-sectional study, we combined data from the 2011–2012 and 2013–2014 NHANES database with 2,537 older adults, which aimed at investigating the associations of LCD score and cognitive performance. After adjusted confounders, the findings displayed that LCD score might be associated with the CERAD word learning subtest and a possible “U-shaped” dose–response relationships were also detected, which indicated that the LCD score was related to the cognitive performance for older adults. These findings might highlight the importance of the higher intakes of fat, protein, and the lower intake of carbohydrate for maintaining specific aspects of cognitive performance among older adults.

The association between cognitive performance and diet has been considered as a hot topic in recent years. Previous researches reported that nutrients of food could regulate the immune system and alter neuroinflammatory processes associated with the pathogenesis of Alzheimer's disease and cognitive impairment [19, 20]. In the study of Wengreen et al., they explored the associations between Dietary Approaches to Stop Hypertension (DASH)-Mediterranean-style dietary patterns and age-related cognitive change, and the result showed that older adults who followed the DASH- Mediterranean-style dietary patterns had higher levels of cognitive function [21]. Furthermore, Coelho-Júnior et al., also pointed out that high adherence to Mediterranean diet was cross-sectionally related to the cognitive function [22]. A ketogenic diet as a very high-fat, low-carbohydrate diet, which was considered as a fasting-like effect putting the body into a state of ketosis [23]. Davis and colleagues expounded that the ketogenic diet may delay, ameliorate, or prevent progression of cognitive decline [24], but older adults with Alzheimer's disease may have difficulty complying with ketogenic diet interventions. LCD, which have a similar composition with the ketogenic diet, have aroused wide concern in the recent years [25].

To the best of our knowledge, this is the first study to have examined the relationship between LCD score and the risk of cognitive performance for older adults based on the NHANES database. Of the three cognitive tests, higher LCD score displayed a protective effect when evaluated by the CERAD word learning subtest, implying the prominent role of LCD score in older adults' ability to learn both immediate and delayed learning ability for new verbal information [26]. In other words, a lower carbohydrate intake and higher fat or protein intake could might be related to a decreased risk of low cognitive performance in moderation. The mechanism about the relationship between LCD score and cognitive performance is unclear, the possible reason was that proper lower carbohydrate intake and high fat and protein intake may cause a state of ketosis, which helped to improve oxidative stress and inflammation, and in turn improved cognitive performance [27]. However, it is worth mentioning that the association of LCD score with AFT, DSST were not statistically significant. The result might indicate that the effect of LCD score on verbal fluency, processing speed, sustained attention and working memory in the older population was less significant. Of course, more research is needed in the future to explain these associations.

In addition, LCD score was associated with the cognitive performance, especially for population who aged < 65 years or BMI 25–30 kg/m^2^ or was married/separated, or had an education level of high school or above. The findings might provide a reference in reducing the risk of low cognitive performance by decreasing the carbohydrate intake and increasing the fat or protein intake for older adults in moderation, especially in population who aged < 65 years or BMI 25–30 kg/m^2^ or was married/separated, or had an education level of high school or above. Additionally, it is worth mentioning that the relationship of LCD score (12–17 points) and cognitive performance was significant in both men and women. However, when the LCD score was between 4–8 points, the relationship was robust only in the older male population. The current literature has not provided a clear explanation for sex differences in the association between LCD score and cognitive performance. This difference between men and women may be due to the fact that men are more affected by LCD scores than women [28, 29]. More prospective studies are needed to further elucidate the sex differences in the association between LCD score and cognitive performance among older adults.

The present study has several advantages. This is the first study to show the associations of LCD score and cognitive performance among a representative sample of the US population aged ≥ 60 years. LCD score are easy to calculate and more practical. Additionally, this study included a relatively large sample size, which makes the conclusions more reliable and makes it possible to perform subgroup analysis according to age, BMI, gender, marital status, education.

Nevertheless, there were some limitations that should be pointed out. Firstly, given that this research designed as a cross-sectional study, we cannot be able to ascertain a causal relationship of LCD score and cognitive performance. Secondly, the 24-h dietary recall interview is based on a questionnaire, which may lead to misclassification of food intake. Lastly, we excluded some participants who had incomplete cognitive tests or had missing information, we couldn't be sure whether these people swayed the results. Thus, the results should be interpreted with caution.

## Conclusion

In summary, we observed that the adherences to LCD score might be associated with the risk of cognitive performance among a representative sample of the US elderly population. The association between LCD score and cognitive performance was stronger in older adults who aged < 65 years or BMI 25–30 kg/m^2^ or was married/separated, or had an education level of high school or above. However, further large-scale cohort studies are needed to test the causal relationship of LCD and cognitive performance.

## Supplementary Information

**Additional file 1:** **Supplemental Table 1.** The criteria for determining the LCD score. **Supplemental Table 2.** The proportion of missing values. **Supplemental Table 3.** The sensitivity analysis before and after deletion of missing values.

## Data Availability

The datasets used and/or analyzed during the current study are available from the NHANES database, https://wwwn.cdc.gov/nchs/nhanes/.

## Abbreviations

DGA: Dietary Guidelines for Americans
NHANES: National Health and Nutrition Examination Survey
CDC: Centers for Disease Control and Prevention
CERAD: Consortium to Establish a Registry for Alzheimer’s Disease
AFT: Animal Fluency Test
DSST: Digit Symbol Substitution Test
