# Supplementary material for: Association of low-carbohydrate-diet score and cognitive performance in older adults: National Health and Nutrition Examination Survey (NHANES)
Source: BMC Geriatr. 2022 Dec 20;22:983. doi: 10.1186/s12877-022-03607-1 (PMC9764565; doi:10.1186/s12877-022-03607-1)
Supplement: Supplementary file 1 — Additional file 1: Supplemental Table 1. The criteria for determining the LCD score. Supplemental Table 2. The proportion of missing values. Supplemental Table 3. The sensitivity analysis before and after deletion of missing values. [file 12877_2022_3607_MOESM1_ESM.docx]

Supplemental Table 1 The criteria for determining the LCD score.

| Points | Carbohydrate/ total energy, % | Protein/ total energy, % | Fat/ total energy, % |
| --- | --- | --- | --- |
| 0 | > 56.0 | <14.1 | <26.0 |
| 1 | 51.6-56.0 | 14.1-15.6 | 26.0-29.5 |
| 2 | 49.1-51.5 | 15.7-16.6 | 29.6-31.6 |
| 3 | 47.1-49.0 | 16.7-17.3 | 31.7-33.2 |
| 4 | 45.2-47.0 | 17.4-18.0 | 33.3-34.7 |
| 5 | 43.3-45.1 | 18.1-18.7 | 34.8-36.1 |
| 6 | 41.2-43.2 | 18.8-19.4 | 36.2-37.7 |
| 7 | 38.8-41.1 | 19.5-20.3 | 37.8-39.5 |
| 8 | 35.4-38.7 | 20.4-21.5 | 39.6-42.0 |
| 9 | 29.3-35 .3 | 21.6-24.0 | 42.1-46.9 |
| 10 | <29.3 | >24.0 | >46.9 |

Note: LCD score is the sum of the scores for the three nutrients, ranging from 0 to 30.

Supplemental Table 2 The proportion of missing values

| Variables | Missing sample size (missing proportion, %) |
| --- | --- |
| Education level | 2 (0.07) |
| Marital status | 3 (0.11) |
| Household income | 108 (3.98) |
| BMI | 35 (1.29) |
| Waistline | 113 (4.17) |
| Diabetes | 2 (0.07) |
| Hypertension | 5 (0.18) |
| Congestive heart failure | 11 (0.41) |
| Coronary heart disease | 15 (0.55) |
| Heart disease | 4 (0.15) |
| Stroke | 5 (0.18) |

Note: BMI, body mass index.

Supplemental Table 3 The sensitivity analysis before and after deletion of missing values

| Variables | After deletion (n=2537) | Before deletion (n=2713) | Statistics | *P* |
| --- | --- | --- | --- | --- |
| Total score, M (Q_1_, Q_3_) | 10.00 (5.00, 15.00) | 10.00 (5.00, 15.00) | Z=0.211 | 0.833 |
| Carbohydrate intake score, M (Q_1_, Q_3_) | 2.00 (1.00, 6.00) | 2.00 (1.00, 6.00) | Z=0.301 | 0.763 |
| Fat intake score, M (Q_1_, Q_3_) | 4.00 (1.00, 7.00) | 4.00 (1.00, 7.00) | Z=0.449 | 0.654 |
| Protein intake score, M (Q_1_, Q_3_) | 2.00 (0.00, 5.00) | 2.00 (0.00, 5.00) | Z=-0.263 | 0.792 |
| Gender, n (%) |  |  | χ^2^=0.015 | 0.902 |
| Male | 1245 (49.07) | 1336 (49.24) |  |  |
| Female | 1292 (50.93) | 1377 (50.76) |  |  |
| Age, years, Mean ± SD | 69.33 ± 6.74 | 69.42 ± 6.76 | t=-0.48 | 0.633 |
| Race, n (%) |  |  | χ^2^=0.466 | 0.977 |
| Mexican American | 214 (8.44) | 233 (8.59) |  |  |
| Other Hispanic | 249 (9.81) | 274 (10.10) |  |  |
| Non-Hispanic White | 1269 (50.02) | 1332 (49.10) |  |  |
| Non-Hispanic Black | 592 (23.33) | 642 (23.66) |  |  |
| Other Race | 213 (8.40) | 232 (8.55) |  |  |
| Education level, n (%) |  |  | χ^2^=0.718 | 0.949 |
| Less Than 9th Grade | 261 (10.29) | 294 (10.84) |  |  |
| 9-11th Grade | 347 (13.68) | 381 (14.05) |  |  |
| High School Grad/GED or Equivalent | 598 (23.57) | 637 (23.50) |  |  |
| Some College or AA degree | 729 (28.73) | 769 (28.37) |  |  |
| College Graduate or above | 602 (23.73) | 630 (23.24) |  |  |
| Marital status, n (%) |  |  | χ^2^=0.371 | 0.996 |
| Married | 1414 (55.74) | 1501 (55.39) |  |  |
| Widowed | 470 (18.53) | 514 (18.97) |  |  |
| Divorced | 374 (14.74) | 393 (14.50) |  |  |
| Separated | 69 (2.72) | 71 (2.62) |  |  |
| Never married | 141 (5.56) | 154 (5.68) |  |  |
| Living with partner | 69 (2.72) | 77 (2.84) |  |  |
| Household income, $, M (Q_1_, Q_3_) | 35000.00 (25000.00, 60000.00) | 35000.00 (25000.00, 60000.00) | Z=0.329 | 0.742 |
| BMI, kg/m^2^, Mean ± SD | 29.12 ± 6.42 | 29.12 ± 6.37 | t=0.04 | 0.966 |
| Waistline, cm, Mean ± SD | 102.05 ± 14.72 | 102.06 ± 14.64 | t=-0.03 | 0.978 |
| Diabetes, n (%) |  |  | χ^2^=0.132 | 0.936 |
| Yes | 582 (22.94) | 633 (23.35) |  |  |
| No | 1837 (72.41) | 1954 (72.08) |  |  |
| Borderline | 118 (4.65) | 124 (4.57) |  |  |
| Hypertension, n (%) |  |  | χ^2^=0.034 | 0.855 |
| Yes | 1578 (62.20) | 1691 (62.44) |  |  |
| No | 959 (37.80) | 1017 (37.56) |  |  |
| Congestive heart failure, n (%) |  |  | χ^2^=0.211 | 0.646 |
| Yes | 173 (6.82) | 193 (7.14) |  |  |
| No | 2364 (93.18) | 2509 (92.86) |  |  |
| Coronary heart disease, n (%) |  |  | χ^2^=0.038 | 0.846 |
| Yes | 233 (9.18) | 252 (9.34) |  |  |
| No | 2304 (90.82) | 2446 (90.66) |  |  |
| Heart disease, n (%) |  |  | χ^2^=0.037 | 0.847 |
| Yes | 220 (8.67) | 239 (8.82) |  |  |
| No | 2317 (91.33) | 2470 (91.18) |  |  |
| Stroke, n (%) |  |  | χ^2^=0.274 | 0.601 |
| Yes | 166 (6.54) | 187 (6.91) |  |  |
| No | 2371 (93.46) | 2521 (93.09) |  |  |
| Magnesium, mg, M (Q_1_, Q_3_) | 263.00 (199.00, 343.50) | 261.50 (197.50, 342.00) | Z=0.424 | 0.672 |
| Calcium, mg, M (Q_1_, Q_3_) | 768.00 (549.00, 1048.00) | 765.00 (547.50, 1040.00) | Z=0.497 | 0.619 |
| Vitamin D, mcg, M (Q_1_, Q_3_) | 3.65 (2.05, 6.05) | 3.65 (2.00, 6.10) | Z=-0.024 | 0.980 |
| Psychotropic medication, n (%) |  |  | χ^2^=0.001 | 0.971 |
| No | 2512 (99.01) | 2686 (99.00) |  |  |
| Yes | 25 (0.99) | 27 (1.00) |  |  |

Note: BMI, body mass index.
